# P2X7 receptor-dependent microglia-astrocyte coupling in Alzheimer's disease: from eATP sensing to synaptic and proteostatic failure

**DOI:** 10.3389/fnagi.2026.1915244

**Published:** 2026-08-17

**Authors:** Yiwen Li, Shiming Liu, Jiao Lan

**Affiliations:** Shenzhen Bao'an Chinese Medicine Hospital, The Seventh Clinical College of Guangzhou University of Chinese Medicine, Shenzhen, Guang Dong, China

**Keywords:** Alzheimer's disease, extracellular ATP, extracellular vesicles, microglia-astrocyte crosstalk, neuroinflammation, P2X7 receptor, proteostasis, synaptic dysfunction

## Abstract

Alzheimer's disease (AD) is characterized not only by amyloid-β and tau pathology but also by progressive failure of multicellular homeostasis. The P2X7 receptor (P2X7R), a low-affinity ATP-gated ion channel preferentially activated in extracellular ATP-rich pathological microenvironments, is well positioned to translate local tissue stress into sustained glial dysfunction. Although P2X7R has traditionally been studied as a microglial inflammasome-associated receptor, its broader significance may lie in coupling microglial activation to astrocytic loss of homeostatic support. In this review, we propose an integrative model in which P2X7R functions as a high-threshold inter-glial transducer through five interconnected axes: extracellular ATP amplification, cytokine relay, extracellular vesicle exchange, convergent synaptic modulation and circuit destabilization, and a coordinated clearance-to-retention switch. Microglial P2X7R activation promotes cytokine and reactive oxygen species production, vesicle and mitochondrial release, inflammatory reprogramming, and impaired phagocytic and lysosomal competence. Astrocytic P2X7R may reinforce this environment through feed-forward ATP release, altered gliotransmission, reactive transformation, extracellular vesicle shedding, and disrupted autophagic and lysosomal processing. We further situate this reciprocal loop within the amyloid plaque niche, where dystrophic neurites, stressed synapses, and reactive glia may create a spatially restricted P2X7R-sensitive ATP microdomain. The resulting cross-glial amplification is proposed to connect neuroinflammation with synaptic destabilization and defective proteostasis. This framework also suggests that brain-penetrant and pharmacologically selective P2X7R antagonists could complement protein-targeted therapies, particularly when guided by functional biomarkers of receptor activity. However, the proposed coupling architecture remains an integrative and testable hypothesis because much of the supporting evidence derives from non-AD models, *in vitro* studies, or correlative human tissue analyses. Cell-type-specific, temporally resolved studies are therefore required to establish whether P2X7R-dependent microglia–astrocyte coupling causally drives AD progression.

## Graphical Abstract

**Figure:**
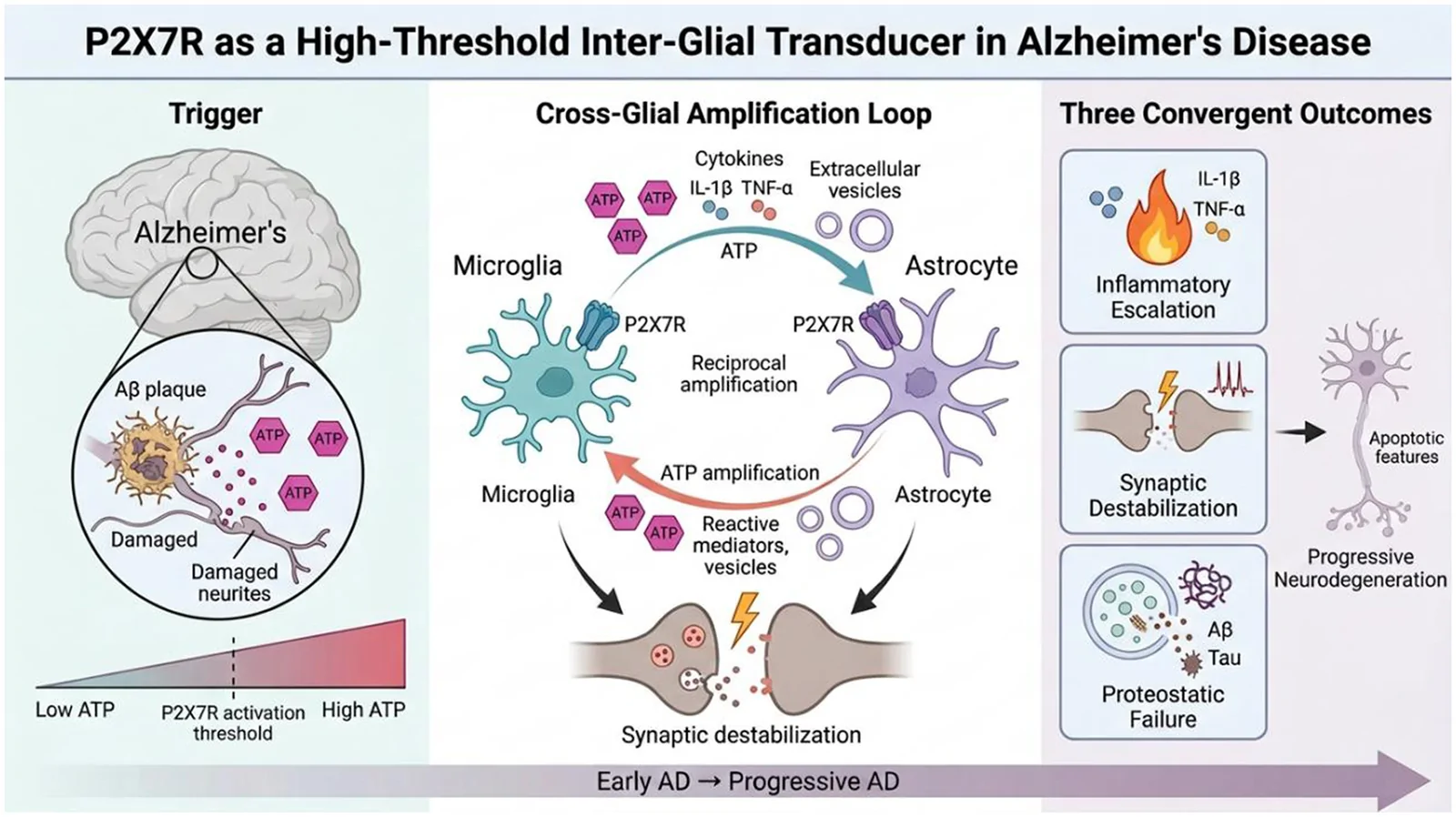
P2X7R as a high-threshold inter-glial transducer in Alzheimer's disease. Lesion-associated extracellular ATP (eATP), released from damaged neurons, dystrophic neurites, and activated glia, may reach concentrations sufficient to activate the low-affinity P2X7 receptor (P2X7R). Microglial P2X7R promotes cytokine and reactive oxygen species production, extracellular vesicle release, inflammatory membrane remodeling, and reduced clearance competence. Astrocytic P2X7R reinforces the altered extracellular environment through feed-forward ATP release, dysregulated gliotransmission, reactive transformation, extracellular vesicle shedding, and impaired proteostatic support. These responses are proposed to form a reciprocal microglia–astrocyte loop organized around five coupling axes: (1) eATP amplification, (2) cytokine relay, (3) extracellular vesicle exchange, (4) convergent synaptic modulation and circuit destabilization, and (5) a coordinated clearance-to-retention switch. Their convergence promotes inflammatory escalation, synaptic destabilization, and defective protein handling. This schematic presents an integrative and testable model rather than an established causal sequence in Alzheimer's disease.

## 1 Introduction

Alzheimer's disease (AD) is commonly described through amyloid plaques and tau aggregates. A fuller account of why neural circuits progressively lose stability, why some brain regions deteriorate earlier than others, and why synaptic dysfunction often precedes massive neuronal loss requires attention to how surrounding cells respond to these lesions. A more useful view is that AD develops when the mechanisms that normally allow different brain cell populations to coordinate their responses to stress begin to fail. In this framework, the most important issue is not only how neurons are injured, but how the multicellular environment surrounding them converts local pathology into self-sustaining tissue dysfunction. Among the signaling systems capable of translating cellular stress into multicellular responses, purinergic signaling is especially relevant because it links metabolic disturbance, membrane damage, inflammatory activation, and synaptic regulation through the same extracellular cue: adenosine triphosphate (ATP).

ATP is released by virtually all cells, and once present in the extracellular space it is rapidly sensed by purinergic receptors and metabolized by neighboring elements of the brain parenchyma (Shigetomi et al., 2023). This degradation is carried out largely by a cascade of ectonucleotidases: the ectonucleoside triphosphate diphosphohydrolase CD39 (NTPDase1) sequentially hydrolyzes ATP to ADP and AMP, and the ecto-5′-nucleotidase CD73 then converts AMP to adenosine, thereby simultaneously terminating the ATP signal and generating an adenosine signal that engages P1 receptors (Zimmermann et al., 2012; Antonioli et al., 2013). Under physiological conditions, extracellular ATP (eATP) is therefore maintained at very low concentrations by the balance between restricted release and this rapid enzymatic degradation. In contrast, damaged neurons, activated glia, mechanical stress, ischemia, hypoxia, oxidative injury, and proteinopathic lesions all increase ATP release into the extracellular compartment (McLarnon, 2017; Morciano et al., 2017). This difference in concentration is not trivial. It determines whether ATP acts as a short-range physiological messenger or as a danger-associated molecular pattern that shifts glial cells into a reactive mode. The receptor most tightly associated with this pathological threshold is the P2X7 receptor (P2X7R), an ATP-gated ion channel distinguished from other purinergic receptors by its low affinity for ATP (half-maximal activation requiring several hundred micromolar to ~1 mm, vs. low-micromolar for other P2X subtypes; see Section 2), prolonged activation profile, and unusually strong coupling to inflammatory and membrane-remodeling pathways (Surprenant et al., 1996; Rassendren et al., 1997). P2X7R was originally identified as the cytolytic “P2Z” receptor and subsequently cloned from rat and human tissue, where it was recognized as the only P2X subunit capable of coupling ATP sensing to membrane permeabilization and cell death.

In AD, the significance of P2X7R cannot be understood adequately by examining microglia and astrocytes as separate compartments. The receptor becomes most important when it links them. Microglia and astrocytes occupy different positions in the diseased brain: microglia are the principal innate immune sentinels and phagocytes (Deng et al., 2024; Pallarés-Moratalla and Bergers, 2024), whereas astrocytes shape neurotransmitter balance, metabolic support, extracellular ion homeostasis, and protein clearance (Araque et al., 2014; Preman et al., 2021). But these roles do not remain independent once amyloid accumulation, neuritic injury, and persistent eATP elevation emerge. Instead, both glial populations enter a reciprocal signaling loop in which microglial inflammatory outputs alter astrocytic state, and astrocytic dysfunction in turn reshapes microglial activation, thereby amplifying pathology. We argue here that P2X7R is a central node in this loop.

This emphasis is important because much of the literature has treated P2X7R primarily as an inflammasome-associated receptor in microglia. That view is partly correct, but too narrow for AD. In the plaque-bearing brain, P2X7R influences not only cytokine release but also extracellular vesicle trafficking, mitochondrial exchange, ATP-dependent feed-forward signaling, phagocytic switching, autophagic competence, gliotransmission, and glial state transitions (Falzoni et al., 2024; Tewari et al., 2024). Its biological relevance therefore lies not in one pathway but in its ability to synchronize several maladaptive processes at once. The receptor is especially suited for this task because it remains relatively quiescent in healthy tissue and becomes strongly engaged only within stressed microdomains where ATP accumulates to abnormal levels (Gordon, 1986; Linden et al., 2019). In other words, by requiring high ATP concentrations, P2X7R functions as a discriminator that reports specifically on a diseased extracellular environment.

Another reason to revisit this receptor in AD is that the debate over its cellular distribution in the central nervous system has often obscured the larger mechanistic question. Early studies identified functional P2X7R expression in microglia (Haas et al., 1996), and subsequent work strengthened the case for pre-dominant expression in microglia, oligodendrocytes, and subsets of astrocytes (Zhao et al., 2021). Some groups have proposed neuronal expression as well (Jimenez-Pacheco et al., 2013; Metzger et al., 2017; Miras-Portugal et al., 2017), but even if neuronal P2X7R is present in certain contexts, the dominant tissue-level actions relevant to chronic neurodegeneration appear to be glial. This matters because a glia-centered interpretation leads to a different therapeutic logic. If P2X7R mainly acts by coupling microglial immune activation to astrocytic failure of homeostatic support, then the receptor should be targeted not simply as an inflammatory channel, but as a mediator of cross-glial disequilibrium.

Several recent reviews have already surveyed P2X7R in neurodegeneration and AD, addressing its structure and pharmacology, its role in neuroinflammation, and its therapeutic potential (Calzaferri et al., 2020; Francistiová et al., 2020; Chen et al., 2021; Zheng et al., 2024; Bedetta et al., 2025). These are valuable, but they are largely organized cell-by-cell or pathway-by-pathway, treating microglial and astrocytic P2X7R as parallel compartments and cataloging downstream effects (inflammasome activation, cytokine release, autophagy) as a list of separable functions. The gap we address is conceptual rather than encyclopedic: none of these reviews frames P2X7R as the coupling node that mechanistically links microglial and astrocytic pathology into a single self-reinforcing loop, nor do they explain how a receptor's high activation threshold specifically converts lesion-associated ATP into reciprocal glial state transitions. This review contributes (a) an explicit inter-glial “missing middle,” defined by five convergent coupling axes; (b) integration of that loop into the amyloid plaque niche alongside TREM2/ApoE-guided engagement; and (c) a therapeutic logic derived from the coupling model rather than from generic inflammation blockade.

We wish to be explicit about the epistemic status of what follows. The coupling model developed here is an integrative hypothesis, not an established consensus. Much of the supporting evidence derives from non-AD systems, *in vitro* preparations, or correlative human tissue analyses, and the causal architecture we propose—that P2X7R links microglial and astrocytic pathology into a single reinforcing loop—has not been directly demonstrated in AD. Throughout, we therefore frame the model as a set of testable predictions and, where relevant, note simpler alternative interpretations that the current data cannot exclude. Accordingly, the purpose of this review is not to catalog every reported effect of P2X7R in isolation. To hold ourselves to this, we adopt a single operational rule throughout: an individual finding is discussed only insofar as it feeds one of the five coupling axes, and effects confined to a single cell type without onward propagation are treated as secondary. On this basis we reorganize the available evidence around an integrated thesis: that in AD, P2X7R may function as a high-threshold inter-glial transducer converting eATP elevation into reciprocal microglial–astrocytic amplification of inflammation, synaptic dysfunction, and defective protein handling. To keep this argument in focus, the review is organized around five coupling axes that together constitute the proposed microglia–astrocyte loop: (1) eATP amplification, (2) cytokine relay, (3) extracellular vesicle exchange, (4) convergent synaptic modulation and circuit destabilization, and (5) a coordinated clearance-to-retention switch. The remainder of the review follows this architecture directly. Section 2 establishes why the receptor's biophysical properties make it a high-threshold sensor. Sections 3 and 4 examine microglial and astrocytic outputs respectively, with each output assigned to one of the five axes rather than cataloged in isolation. Section 5 assembles these outputs into the reciprocal loop, treating the five axes in turn: eATP amplification (Section 5.1), cytokine relay (Section 5.2), extracellular vesicle exchange (Section 5.3), convergent synaptic modulation and circuit destabilization (Section 5.4), and the coordinated clearance-to-retention switch (Section 5.5). Sections 6–8 then situate the loop within the amyloid plaque niche and trace its two principal downstream consequences—synaptic dysfunction (Section 7) and proteostatic failure (Section 8). Section 9 develops the therapeutic logic that follows from the coupling model. Throughout, we return to the same five axes so that the reader can locate each piece of evidence within the loop.

## 2 Why P2X7R is ideally positioned to couple glial responses in AD

Purinergic signaling is often introduced as a receptor family taxonomy, but in AD its pathophysiological significance lies in threshold detection. P1 receptors respond to adenosine and generally shape modulatory programs under relatively low ligand concentrations (Shigetomi et al., 2023). P2Y receptors, which are metabotropic, detect ATP, ADP, UTP, or UDP and regulate migration, phagocytosis, and inflammatory signaling in glia (Madry et al., 2018; von Kügelgen, 2019; Illes et al., 2020). P2X receptors are ATP-gated ion channels that mediate rapid ionic fluxes (Valera et al., 1994). Among them, P2X7R is unusual because it remains comparatively insensitive to ATP and is therefore preferentially engaged in conditions of intense cellular stress (Surprenant et al., 1996; North, 2002; Liu et al., 2024). The defining physiological properties of the receptor—its requirement for high ATP concentrations for half-maximal activation, its slow desensitization, and its weak desensitization and its association, during sustained activation, with membrane permeabilization and altered permeability to larger solutes—were established in the earliest functional characterizations of the cloned receptor and remain the basis for its role as a sensor of pathological ATP accumulation (Surprenant et al., 1996; North, 2002). The magnitude of this concentration requirement is best appreciated in comparison with the surrounding purinergic system. Under physiological conditions, resting eATP in the brain parenchyma is maintained in the low nanomolar range (on the order of ~1–10 nM), and most other ionotropic P2X subunits (P2X1–P2X6) are half-maximally activated by low-micromolar ATP (roughly 1–10 μm) (North, 2002). P2X7R is distinguished by an EC50 for ATP that is one to two orders of magnitude higher—typically in the range of several hundred micromolar to ~1 mm, with near-maximal current requiring low-millimolar ATP (Surprenant et al., 1996; North, 2002). This apparent affinity is further shifted rightward by physiological divalent cations, because the tetra-anionic species ATP4^+^ rather than Mg^2+^- or Ca^2+^-bound ATP is the true agonist; in the presence of normal extracellular Mg^2+^/Ca^2+^, the effective threshold for meaningful P2X7R activation therefore lies well into the high-micromolar-to-millimolar domain. It is precisely this ~10^4^-10^6^-fold gap between resting eATP and the P2X7R activation threshold that allows the receptor to remain comparatively quiescent during routine purinergic signaling and to report selectively on lesion-associated ATP accumulation. This low sensitivity is the basis of the receptor's biological specialization. By requiring high eATP, P2X7R avoids interference with routine signaling and is engaged only within lesion-associated microenvironments—in AD, the plaque-associated neurites, metabolically stressed synapses, activated glia, and damaged membranes that serve as local ATP sources (Shigetomi et al., 2023). It thus operates as a molecular discriminator of pathological microdomains where homeostatic signaling has escalated into danger signaling, precisely the spatial pattern that makes it so influential in chronic neurodegeneration.

Structurally, P2X7R differs from other P2X family members in ways that reinforce this role. The receptor forms a trimeric ATP-gated channel like other P2X receptors, but its long intracellular C-terminus enables a wider range of interactions with signaling and trafficking machinery (Liu et al., 2024). Cryo-electron microscopy has shown that this cytoplasmic extension contains structural features explaining the receptor's weak desensitization and sustained functional profile (Oken et al., 2024b)—properties essential for converting chronically elevated ATP in AD lesions into lasting cellular state changes rather than transient ionic events.

**Figure 1:**
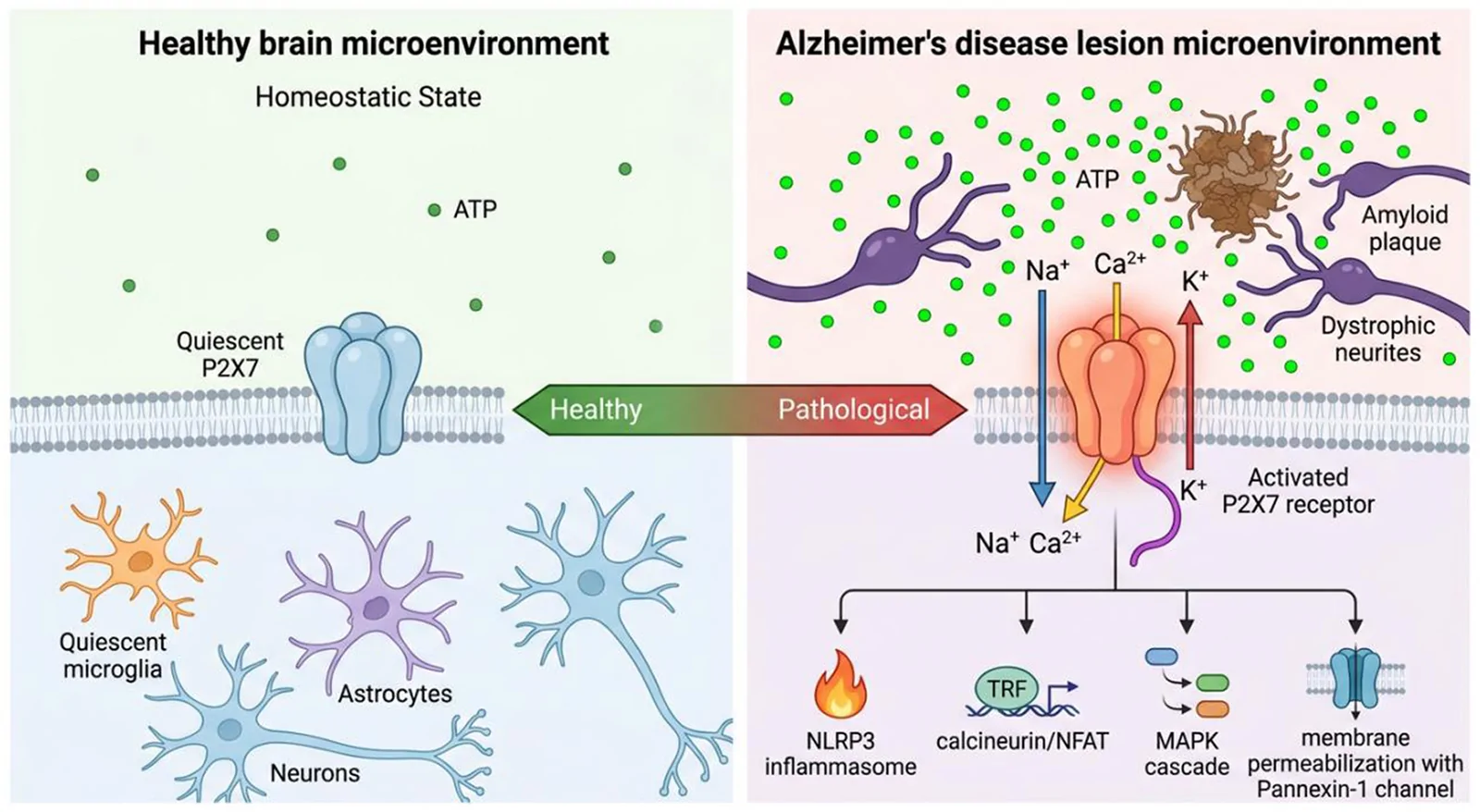
Structural and functional features of P2X7R as a high-threshold extracellular ATP sensor. P2X7R is a trimeric ATP-gated cation channel distinguished by its relatively low ATP sensitivity, weak desensitization, and long intracellular C-terminal domain. Whereas resting extracellular ATP (eATP) in brain parenchyma is generally maintained in the low-nanomolar range and most other P2X receptors respond to low-micromolar ATP, substantial P2X7R activation typically requires high-micromolar-to-millimolar ATP concentrations. This activation threshold favors receptor engagement in lesion-associated microenvironments rather than during routine purinergic signaling. Channel opening permits Na^+^ and Ca^2^^+^ influx and K^+^ efflux. The resulting Ca^2^^+^ elevation activates transcriptional and signaling pathways, including calcineurin/NFAT and MAPK-related cascades, whereas K^+^ loss generates an ionic environment permissive for inflammasome activation. Sustained stimulation can additionally promote membrane remodeling, P2X7R-associated Pannexin-1 signaling, and feed-forward ATP release. The relative contributions of the P2X7R channel, receptor-associated membrane pathways, and Panx1 to large-molecule permeability remain context dependent. Together, these properties enable P2X7R to convert intense extracellular stress into prolonged inflammatory and intercellular signaling responses. eATP, extracellular adenosine triphosphate; NFAT, nuclear factor of activated T cells; MAPK, mitogen-activated protein kinase; NLRP3, NOD-like receptor protein 3; Panx1, Pannexin-1.

Its downstream consequences extend beyond cation influx. P2X7R activation permits Na^+^/Ca^2+^ entry and K^+^ exit (Burnstock and Kennedy, 2011); the Ca^2+^ rise engages transcriptional cascades (calcineurin/NFAT, MAPK, CaMKII/CREB) that regulate inflammatory gene expression, survival, and remodeling, while K^+^ loss creates inflammasome-permissive conditions (Ferrari et al., 1999; Bianco et al., 2006). P2X7R therefore acts as a trigger for transcriptional and membrane state transitions, extending its function well beyond ion conduction (Figure 1).

The long-standing discussion about membrane permeabilization and pannexin-1 (Panx1) is also informative in this regard. P2X7R activation has been associated with the passage of larger hydrophilic solutes, ATP release, and signaling through Panx1 in several immune cell types (Pelegrin and Surprenant, 2006, 2009; Di Virgilio et al., 2018). Whether this occurs through the receptor pore itself, through recruited secondary pathways, or through both depending on cell context remains debated. For AD, however, the precise route is less important than the functional implication: once activated strongly enough, P2X7R does not simply open and close. It can change the permeability properties of the membrane, promote eATP amplification, and reorganize communication between neighboring cells. This makes it highly suitable for glial coupling in a chronically stressed tissue.

A final point deserves emphasis. The pathological significance of P2X7R extends well beyond IL-1β release to a broader systems-level function. In glial cells, P2X7R can influence cytokine release, oxidative stress, microvesicle shedding, phagocytosis, autophagy, transmitter release, mitochondrial transfer, and cell survival (Bedetta et al., 2025). Taken together, these effects suggest that P2X7R acts as a state-transition receptor. It marks the moment when eATP no longer signals local activity but instead forces glial cells into a new operating regime. AD is precisely the kind of disease in which such a transition becomes chronic.

## 3 Microglial P2X7R in AD: from immune surveillance to inflammatory propagation

Microglia are the brain's principal innate immune sentinels and rely on a broad purinergic repertoire—adenosine, P2Y, and P2X receptors—to monitor the parenchyma and respond to extracellular nucleotides (Liu et al., 2024; Pallarés-Moratalla and Bergers, 2024). Within this repertoire, P2X7R is distinct in being recruited only under severe ATP accumulation rather than during baseline surveillance, which is what makes it the relevant node when microglial function shifts from surveillance to inflammatory propagation. Our aim in this section is not to inventory microglial P2X7R functions but to establish which microglial outputs feed the inter-glial loop developed in Section 5. Accordingly, each effect below is examined specifically for its capacity to act beyond the activated microglial cell—that is, to alter the astrocytic state or the shared extracellular environment. Read this way, the microglial outputs map onto the coupling axes formalized in Section 5: ATP and membrane-remodeling responses may contribute to eATP amplification; soluble mediators and ROS feed the cytokine-relay axis; microvesicle and mitochondrial release contribute to extracellular vesicle exchange; vesicle-mediated effects on neuronal excitability feed convergent synaptic modulation and circuit destabilization; and changes in phagocytic, autophagic, and lysosomal competence contribute to the coordinated clearance-to-retention switch. Effects that do not propagate to neighboring glia are, for our purposes, secondary.

The strongest evidence for functional P2X7R expression in the brain remains in microglia. Microglial P2X7R is detectable during development (Rigato et al., 2012), indicating that the receptor is not merely a late-stage inflammatory addition to the glial membrane. However, in adult pathological tissue its role becomes especially evident. The involvement of P2X7R in AD was first established by primary studies demonstrating that receptor expression is markedly up-regulated around amyloid plaques: P2X7R was shown to be increased in microglia of an APP-transgenic mouse model and to mediate amyloid-β-induced superoxide production (Parvathenani et al., 2003), while parallel work confirmed elevated P2X7R immunoreactivity in human AD brain and in Aβ-injected rat hippocampus (McLarnon et al., 2006). Subsequent functional work established that microglial activation by amyloid-β itself requires P2X7R expression (Sanz et al., 2009). These converging genetic, human-tissue, and functional data ground the receptor's disease relevance in primary evidence rather than in expression correlation alone. This localization is biologically plausible because plaque-associated regions bring activated microglia into close proximity to damaged neurites and potential sources of extracellular ATP. In such lesions, P2X7R becomes a mechanism through which microglia translate local tissue injury into inflammatory output.

### 3.1 Cytokine and ROS output: feeding the cytokine-relay axis

One of the best-characterized microglial consequences of P2X7R activation is NLRP3 inflammasome signaling. In LPS-primed murine macrophages and microglia, ATP acting on P2X7R promotes caspase-1–dependent maturation and release of IL-1β and IL-18 (Mariathasan et al., 2004; Ferrari et al., 2006). The classical model attributes this to K^+^ efflux downstream of P2X7R, but recent work in mouse macrophages implicates additional K^+^ channels (TWIK-2, THIK-1) in generating the ionic environment for inflammasome assembly (Di et al., 2018), and P2X7R activation in human macrophages can drive IL-1β release through an NLRP3-independent route (Bockstiegel et al., 2023). These findings—largely from primed peripheral or immortalized cells rather than AD tissue—suggest that P2X7R acts less as an obligatory NLRP3 trigger than as an upstream organizer of the membrane-signaling platform permissive for inflammasome activation (Orioli et al., 2017); the extent to which this operates in plaque-associated human microglia remains to be established directly.

P2X7R activation also stimulates ROS production: in cultured rat spinal astrocytes, BzATP-driven P2X7R signaling generated ROS that contributed to IL-6 release (Munoz et al., 2020). Although demonstrated in spinal astrocytes rather than forebrain microglia, this illustrates a P2X7R–ROS–cytokine axis that, superimposed on the mitochondrial and redox stress already present in AD, would be expected to compound local damage. Cytokines and ROS released from P2X7R-activated microglia affect not only neurons but also neighboring astrocytes. That is a point often underemphasized in receptor-centered discussions. The immediate output of microglial P2X7R is not simply “inflammation”; it is a biochemical environment that imposes a new reactive state on surrounding glia.

### 3.2 Microvesicle and mitochondrial release: feeding the eATP-amplification and extracellular-vesicle-exchange axes

P2X7R also reshapes microglial biology through membrane remodeling. *In vitro*, ATP/BzATP stimulation of P2X7R in murine microglia and human monocytes triggered rapid (within minutes) shedding of plasma-membrane microvesicles carrying bioactive IL-1β, dependent on p38 MAPK and acid sphingomyelinase (MacKenzie et al., 2001). These vesicles package bioactive mediators for targeted delivery (Turola et al., 2012). Functionally, microglia-derived microvesicles applied to cultured hippocampal neurons increased miniature excitatory post-synaptic current frequency via enhanced sphingolipid metabolism (Antonucci et al., 2012), linking innate immune activation to circuit-level excitability—though this was shown in reduced neuronal cultures rather than in AD networks. These findings indicate that microglial P2X7R activation generates diffusible and vesicle-packaged mediators capable of acting on cells beyond the activated microglia themselves.

A more provocative aspect concerns horizontal transfer of organelles and signaling components. In cultured mouse microglia, P2X7R was identified as a master regulator of microparticle and mitochondria release and exchange, such that recipient cells acquired donor-derived material (Falzoni et al., 2024); intercellular mitochondrial transfer has separately been documented between neural cells and astrocytes (Gao et al., 2019; English et al., 2020). These studies were performed *in vitro* and not in AD tissue, so their contribution to spatial expansion of inflammatory competence around plaques remains a hypothesis rather than a demonstrated *in vivo* mechanism. In AD, where reactive microglia cluster around lesions rather than remaining evenly distributed, such horizontal transmission could help explain how inflammatory competence expands through a local glial territory. Through this transfer of microparticles and mitochondria, P2X7R-dependent signaling competence may be conferred on recipient cells that were not themselves directly activated.

### 3.3 The phagocytic-to-inflammatory switch: feeding the coordinated clearance-to-retention axis

Another dimension of microglial P2X7R is its ability to regulate phagocytosis in opposite directions depending on receptor state (Karasawa and Kawate, 2016; Allsopp et al., 2017). In transfected HEK293 cells and murine macrophages/microglia, unliganded P2X7R supported uptake of apoptotic cells and beads, functioning as a scavenger receptor in the absence of ATP (Gu et al., 2011). Conversely, ATP occupancy uncouples the receptor from this phagocytic mode and biases microglia toward endolysosomal and inflammatory signaling (Campagno and Mitchell, 2021). This state-dependent, largely *in vitro* behavior implies that chronic peri-plaque ATP could lock microglia into the inflammatory configuration at the expense of clearance—a prediction not yet tested directly in AD tissue. This dual behavior may be highly relevant in AD. Microglia surrounding plaques must balance clearance of extracellular debris with the risk of overactivation. If chronic ATP exposure locks P2X7R into its inflammatory configuration, plaque-associated microglia may gradually lose the phagocytic efficiency needed for tissue containment while gaining the cytokine and ROS output that accelerates damage. In that case, P2X7R would not merely correlate with reactive microgliosis; it would help drive the transition from adaptive clearance to maladaptive inflammation.

### 3.4 Autophagic and lysosomal competence: feeding the coordinated clearance-to-retention axis

Autophagy provides another mechanism through which this transition may occur. In immortalized/primary mouse microglia, brief P2X7R activation transiently raised LC3-II, whereas sustained activation impaired AMPK-dependent mitochondrial and lysosomal function and reduced degradative capacity (Sekar et al., 2018); a parallel effect on autophagy was seen in microglia from SOD1-G93A (ALS-model) mice (Fabbrizio et al., 2017). Because these data derive from *in vitro* microglia and an ALS rather than AD model, their extrapolation to amyloid-associated proteostasis should be regarded as mechanistically plausible but not yet directly validated in AD. This shift from productive degradation to lysosomal dysfunction is highly relevant to proteinopathy. Although much of the mechanistic work was not performed specifically in AD tissue, the implication is clear: a receptor activated by persistent eATP may progressively convert microglia from cells capable of clearing pathological material into cells that process it inefficiently and export inflammatory consequences to the extracellular space.

These findings are particularly meaningful when considered alongside disease-associated microglia (DAM) biology. Microglia in neurodegenerative conditions downregulate some homeostatic markers while upregulating genes linked to lysosomal function, lipid metabolism, and phagocytosis (Cheng and Ho, 2025). TREM2 and ApoE are critical drivers of this transition (Zhong et al., 2018; Jain et al., 2023). In AD, such changes likely begin as an attempt to contain protein aggregates and tissue injury. Our view is that P2X7R does not replace TREM2- or ApoE-driven reprogramming, but overlays it with a high-ATP stress axis. In other words, TREM2 may tell microglia that abnormal material is present, whereas P2X7R may tell them that the extracellular environment has become dangerous enough to justify inflammatory escalation. This distinction may explain why microglia around plaques can display both clearance-associated and damaging features at the same time.

**Figure 2:**
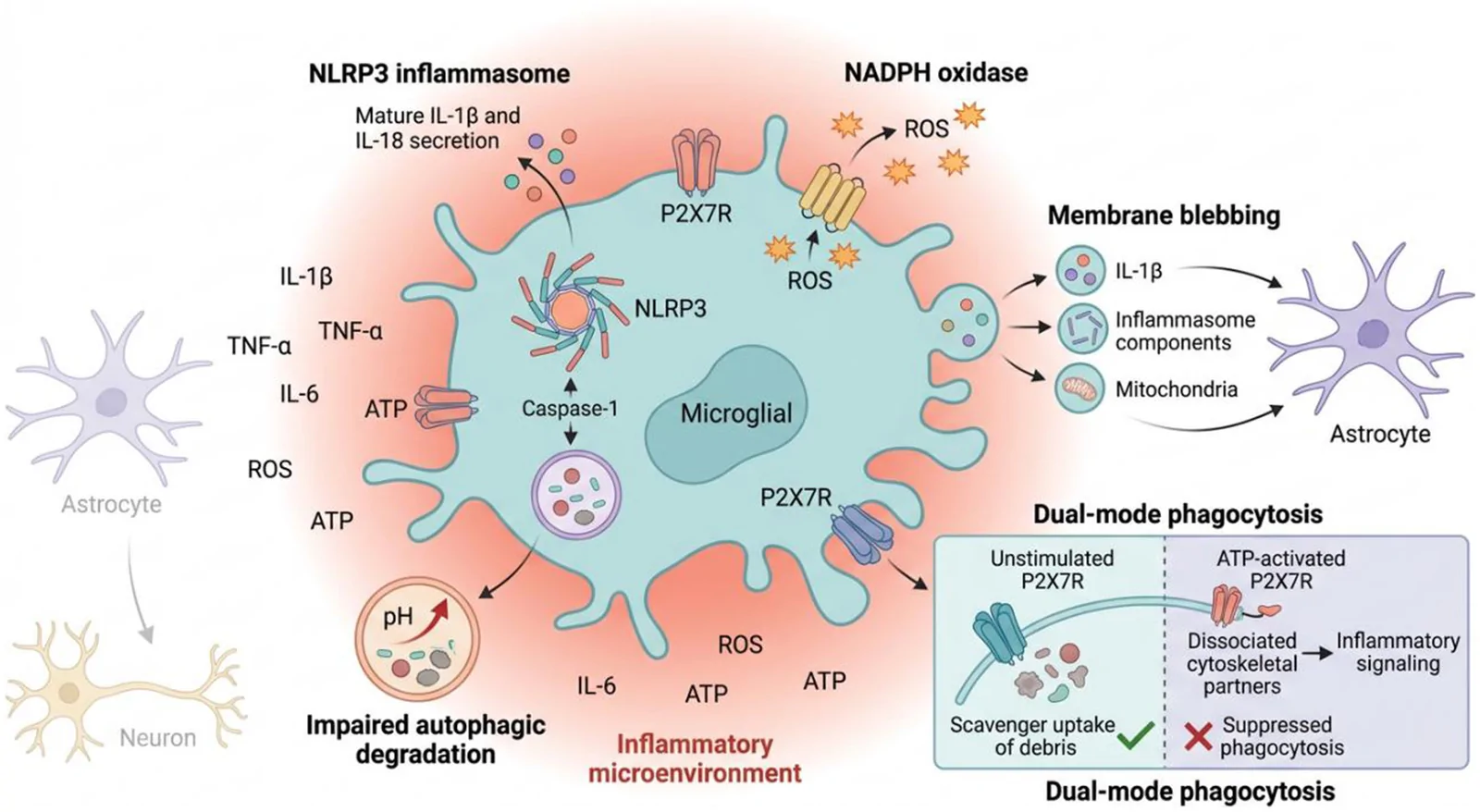
Microglial P2X7R outputs contributing to cross-glial pathological propagation in Alzheimer's disease. In ATP-rich lesion environments, sustained activation of microglial P2X7R shifts microglial activity from homeostatic surveillance and adaptive containment toward inflammatory and secretory responses. P2X7R-associated ionic changes facilitate inflammasome-related signaling and the maturation or release of inflammatory cytokines, including IL-1β and IL-18, although the requirement for NLRP3 varies across species and experimental systems. Receptor activation also promotes reactive oxygen species (ROS) production, generating an extracellular environment capable of inducing reactive changes in neighboring astrocytes. Membrane remodeling downstream of P2X7R supports the shedding of IL-1β-containing microvesicles and other extracellular particles and may facilitate microparticle and mitochondrial exchange. In parallel, receptor state influences microglial housekeeping: unliganded P2X7R can support scavenger-like phagocytosis, whereas sustained ATP-dependent activation favors inflammatory and endolysosomal signaling. Prolonged stimulation may impair mitochondrial, lysosomal, and autophagic function, thereby reducing effective degradation of pathological material. These outputs feed the cytokine-relay, extracellular-vesicle-exchange, ATP-amplification, and clearance-to-retention axes of the proposed microglia–astrocyte loop. Although the individual mechanisms are supported by experimental studies, their coordinated operation in Alzheimer's disease has not yet been directly established. IL-1β, interleukin-1β; IL-18, interleukin-18; ROS, reactive oxygen species; EVs, extracellular vesicles; NLRP3, NOD-like receptor protein 3.

The synthesis that emerges is the organizing claim of this review: none of the microglial outputs above is self-contained. ATP, cytokines and ROS, and vesicle- or mitochondria-associated cargo map primarily onto the eATP-amplification, cytokine-relay, and extracellular-vesicle-exchange axes, respectively. Through downstream effects on neuronal excitability and glial degradative competence, these outputs may also contribute to convergent synaptic modulation and circuit destabilization and to the coordinated clearance-to-retention switch. Microglial P2X7R is therefore best understood not as a list of inflammatory functions but as the first half of a cross-glial transducer whose downstream target is the astrocyte (Figure 2).

## 4 Astrocytic P2X7R in AD: when homeostatic support becomes pathological amplification

Astrocytes regulate glutamate clearance, K^+^ buffering, metabolic support, and protein handling, and in AD they are not passive responders but active determinants of whether local pathology remains contained or spreads (Araque et al., 2014; Lia et al., 2023). P2X7R is increasingly implicated in the transition from this homeostatic role to pathological amplification. As in Section 3, we treat astrocytic P2X7R outputs not as an independent catalog but as the return arm of the coupling loop. The question throughout is whether a given astrocytic response feeds back onto the shared microenvironment or onto microglia themselves. On this criterion, the astrocytic effects discussed below resolve into the same five axes: ATP release through P2X7R-associated Panx1 signaling contributes to eATP amplification; reactive cytokine output feeds the cytokine-relay axis; vesicle shedding contributes to extracellular vesicle exchange; gliotransmitter release feeds convergent synaptic modulation and circuit destabilization; and impaired autophagic or lysosomal processing contributes to the coordinated clearance-to-retention switch. Where an astrocytic finding cannot yet be tied to one of these axes, we say so explicitly.

Compared with microglia, astrocytic P2X7R has been more difficult to characterize, in part because expression appears heterogeneous and because strong ATP elevations are usually required for clear receptor activation (Beltran-Lobo et al., 2023). Reporter-based approaches indicate that P2X7R is present in subsets of astrocytes rather than uniformly across all brain regions (Kaczmarek-Hajek et al., 2018). This heterogeneity is often treated as a complication, but in AD it may actually be mechanistically revealing. Not every astrocyte needs to express high levels of P2X7R for the receptor to matter; it is sufficient that P2X7R-positive astrocytes occupy key pathological niches, such as peri-plaque territories, metabolically stressed hippocampal fields, or regions of intense glial crosstalk.

### 4.1 ATP-dependent feed-forward release: feeding the eATP-amplification axis

One reason astrocytic P2X7R is relevant to AD is its role in ATP-dependent feed-forward signaling. Astrocytes release ATP through exocytotic and non-exocytotic pathways (Harada et al., 2015), and the P2X7R-Panx1 complex has been identified as an important ATP-release route in spinal cord astrocytes (Garré et al., 2010). This organization has profound implications. A receptor activated by eATP can help generate additional eATP, thereby converting a local stress cue into a self-amplifying field signal. In an AD lesion, where neuritic dystrophy and glial activation already elevate ATP, astrocytic P2X7R could therefore function as an amplifier rather than merely a responder.

### 4.2 Reactive transformation and cytokine output: feeding the cytokine-relay axis

A second major contribution of astrocytic P2X7R in AD concerns inflammatory transformation. Astrocytes respond to eATP with intracellular Ca^2+^ elevations and intercellular signaling waves (Beltran-Lobo et al., 2023). While lower-affinity receptors probably dominate under physiological ATP concentrations, P2X7R becomes more relevant in high-ATP environments or when receptor sensitivity is altered. Indeed, nicotinamide adenine dinucleotide (NAD^+^)-dependent ADP-ribosylation mechanisms known to sensitize P2X7R in peripheral cells may also operate in astrocytes under ischemia-related conditions (Hirayama and Koizumi, 2017; Hirayama et al., 2021). Although this pathway was not originally described in AD, it is highly relevant conceptually because it shows that receptor activity cannot be inferred solely from eATP concentration. Disease-associated changes in the astrocytic membrane environment may lower the effective threshold for P2X7R engagement.

Astrocytic P2X7R can couple stress to inflammasome-related cytokine output. In cultured and *ex vivo* rat optic-nerve-head astrocytes, mechanical strain elevated IL-1β and primed NLRP3 through sequential pannexin-1–dependent ATP release and P2X7R activation (Beckel et al., 2014; Albalawi et al., 2017), and in rodent ocular-hypertension models P2X7R stimulation induced pan-, A1-, and A2-type reactive astrocyte gene programs (Campagno et al., 2024). The originating stimulus here is mechanical strain, not amyloid, but the principle is transferable: astrocytic P2X7R can convert diverse extracellular stresses into a cytokine-producing reactive phenotype. In the AD brain, astrocytes are continually exposed not only to ATP but also to microglial cytokines, misfolded proteins, and altered neuronal activity, and under these conditions P2X7R likely acts as a convergence point at which these signals are translated into reactive astrogliosis.

### 4.3 Vesicle shedding: feeding the extracellular-vesicle-exchange axis

Extracellular vesicle biology extends this interpretation. ATP stimulation increases extracellular vesicle release from activated astrocytes, and this depends on P2X7R (Abdullah et al., 2024). The cargo and functional significance of these vesicles in AD remain incompletely defined, but it is difficult to imagine that such a process is biologically neutral in a proteinopathic brain. Astrocytic vesicles may distribute inflammatory mediators, metabolic enzymes, proteases, or receptor-modifying molecules to neighboring glia and neurons. At a minimum, their release indicates that astrocytic P2X7R participates in a broader secretory remodeling program.

### 4.4 Gliotransmitter release: feeding convergent synaptic modulation and circuit destabilization

Astrocytic P2X7R can engage gliotransmission. In cultured rat astrocytes, ATP/BzATP triggered release of glutamate and aspartate (Duan et al., 2003); in rat hippocampal slices, astrocyte-derived glutamate acting on extrasynaptic NMDA receptors drove tonic currents and neuronal synchrony (Fellin et al., 2004, 2006); and in rat hippocampal preparations, high BzATP promoted astrocytic GABA release, shifting excitation–inhibition balance (Zhang et al., 2022). The ATP-release route itself has been mapped to a P2X7R–Panx1 complex in cultured spinal-cord astrocytes stimulated with FGF-1 (Garré et al., 2010). These are *ex vivo*/*in vitro* rodent findings using pharmacological agonists rather than AD models; nonetheless, because early AD networks show aberrant hyperexcitability, an astrocytic mechanism mobilizing transmitter output in ATP-rich microdomains is directly relevant to prodromal disease.

The interpretation of D-serine-related findings requires greater caution. Studies have shown release of D-serine through a P2X7R-Panx1-associated mechanism in astrocytes (Pan et al., 2015), but more recent work suggests that mature astrocytes may contribute mainly L-serine, which neurons then use for D-serine production (Wolosker et al., 2016). This revision does not reduce the importance of astrocytes in synaptic plasticity; it refines the metabolic route through which they act. In AD, where aerobic glycolysis and astrocytic serine metabolism are compromised early (Le Douce et al., 2020), P2X7R-related metabolic disturbance would feed the synaptic-modulation axis (Section 5.4) from a second direction—not only through gliotransmitter release but by degrading the metabolic substrates required for synaptic maintenance.

### 4.5 Protein handling: feeding the coordinated clearance-to-retention axis

Finally, astrocytes in AD are major handlers of extracellular proteins, engulfing Aβ, internalizing tau species, and relying on lysosomal, ApoE-, LRP1-, and TFEB-related pathways for clearance (Preman et al., 2021). Although the direct role of P2X7R here has not been fully mapped, its documented influence on astrocytic autophagy (examined in Section 8) suggests that chronic ATP exposure could bias astrocytes toward secretory, reactive activity at the expense of degradation—eroding one of their most important protective roles in AD.

**Figure 3:**
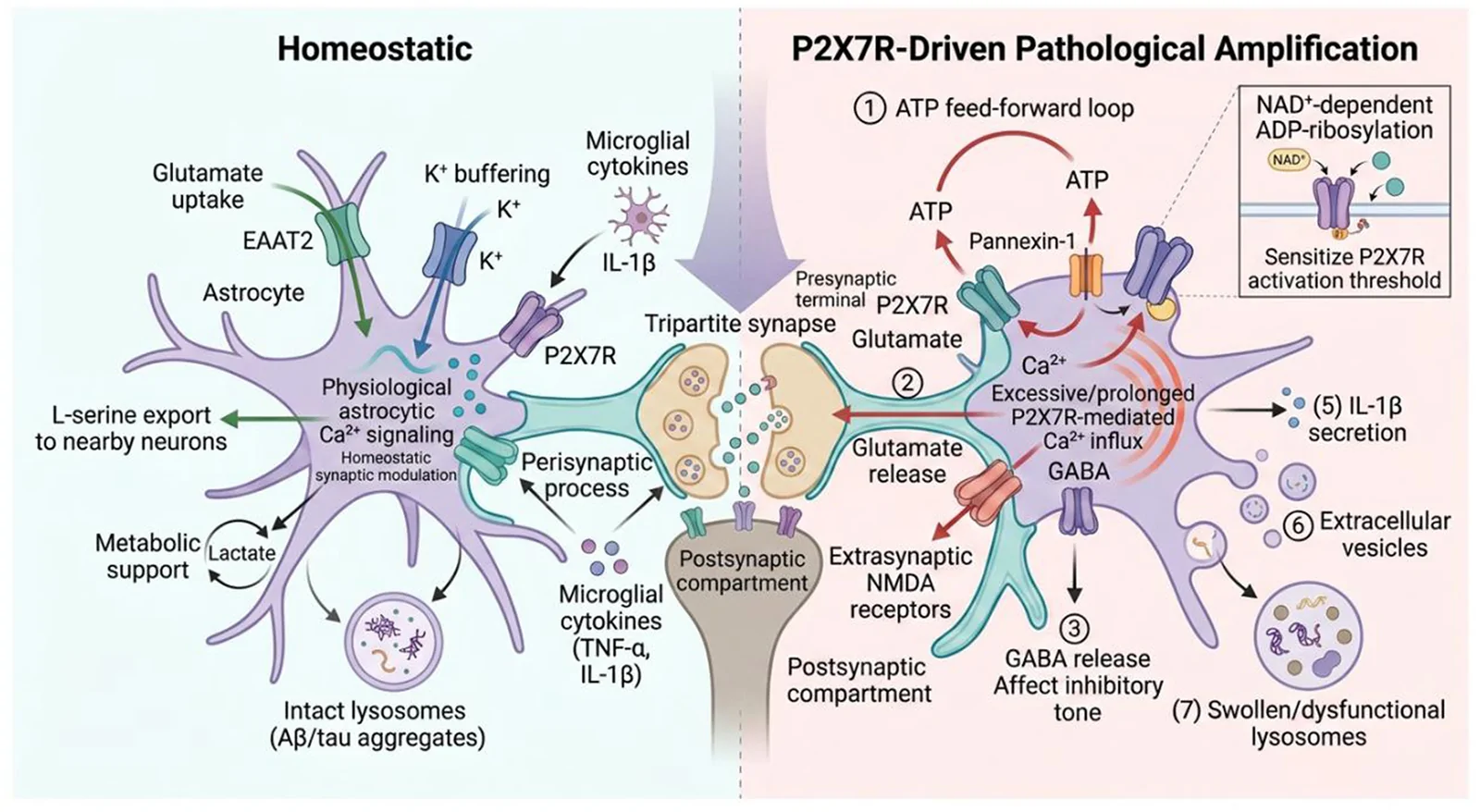
P2X7R-associated conversion of astrocytes from homeostatic regulators to pathological amplifiers. Under physiological conditions, astrocytic perisynaptic processes interact with both pre-synaptic and post-synaptic neuronal compartments and support synaptic homeostasis through EAAT2-mediated glutamate uptake, extracellular K^+^ buffering, metabolic and lactate support, L-serine provision, and lysosomal processing of internalized proteins. Astrocytes also exhibit physiological, spatially restricted Ca^2+^ signals that contribute to normal intercellular communication and synaptic regulation; astrocytic Ca^2+^ signaling is therefore not inherently pathological. In high-ATP environments, sustained P2X7R activation may instead produce excessive or prolonged Ca^2+^ influx and promote a broader pathological secretory program. The principal illustrated outputs include: (1) P2X7R/Pannexin-1-associated feed-forward ATP release; (2) glutamate release and activation of extrasynaptic NMDA receptors; (3) GABA release and altered inhibitory tone; (4) IL-1β secretion; (5) extracellular vesicle release; and (6) impaired autophagic or lysosomal processing. Microglia-derived cytokines, including TNF-α and IL-1β, may further reinforce astrocyte reactivity. NAD^+^-dependent ADP-ribosylation is shown as a potential sensitizing mechanism that may lower the effective threshold for P2X7R activation under specific stress conditions. Together, these changes feed the ATP-amplification, synaptic-modulation, cytokine-relay, extracellular-vesicle-exchange, and clearance-to-retention axes of the proposed cross-glial loop. Evidence for the individual pathways originates from multiple experimental contexts, and their coordinated operation in Alzheimer's disease astrocytes remains to be directly demonstrated. EAAT2, excitatory amino acid transporter 2; Panx1, Pannexin-1; NMDA, N-methyl-D-aspartate; GABA, γ-aminobutyric acid; EVs, extracellular vesicles; NAD^+^, nicotinamide adenine dinucleotide.

For this reason, astrocytic P2X7R may contribute to the conversion of homeostatic astrocytes into pathological amplifiers through five interconnected processes: eATP amplification, cytokine relay, extracellular vesicle exchange, convergent synaptic modulation and circuit destabilization, and declining autophagic or lysosomal competence as part of the coordinated clearance-to-retention switch. That transformation becomes particularly damaging when it occurs in dialogue with activated microglia (Figure 3).

## 5 The missing middle: how P2X7R connects microglia and astrocytes rather than acting in parallel

A recurring limitation in the literature is that microglial and astrocytic P2X7R are often discussed in separate sections, as if each cell type responded independently to ATP. In AD, this separation is artificial. The most important pathogenic consequences of P2X7R likely emerge not from receptor activation in either microglia or astrocytes alone, but from the reciprocal loop established between them. That loop is proposed to be sustained by five interconnected coupling axes: (1) eATP amplification, (2) cytokine relay, (3) extracellular vesicle exchange, (4) convergent synaptic modulation and circuit destabilization, and (5) a coordinated clearance-to-retention switch involving changes in phagocytic, autophagic, and lysosomal competence. The pivotal experimental studies underlying these axes, with their model systems, manipulations, key results, and caveats, are summarized in Table 1.

**Table 1:** Pivotal experimental studies underpinning the P2X7R cross-glial model.

Study	Model/species	Manipulation	Key result	Caveat
MacKenzie et al. (2001)	Murine microglia/human monocytes, *in vitro*	ATP/BzATP	Rapid shedding of IL-1β microvesicles	Not AD tissue
Antonucci et al. (2012)	Microglial MVs → rat hippocampal neurons, *in vitro*	Apply MVs	↑ mEPSC frequency (sphingolipid-dependent)	Reduced culture system
Falzoni et al. (2024)	Mouse microglia, *in vitro*	P2X7R activation	Microparticle + mitochondria exchange	*In vitro* only
Gu et al. (2011)	HEK293/macrophages, *in vitro*	No ATP (unliganded)	P2X7R acts as scavenger receptor	State-dependent; not AD
Sekar et al. (2018)	Mouse microglia, *in vitro*	Sustained P2X7R activation	Impaired lysosomal/mitochondrial function	*In vitro*
Fabbrizio et al. (2017)	SOD1-G93A mouse microglia	P2X7R activation	Modulated autophagy	ALS, not AD
Duan et al. (2003)	Rat astrocyte culture	ATP/BzATP	Glutamate/aspartate release	*In vitro*
Fellin et al. (2004, 2006)	Rat hippocampal slices	Astrocytic P2X7R	Extrasynaptic NMDA tonic currents	*Ex vivo*
Garré et al. (2010)	Rat spinal astrocytes, *in vitro*	FGF-1	ATP release via P2X7R–Panx1	Non-AD stimulus
Beckel et al. (2014); (Albalawi et al. 2017)	Rat optic-nerve astrocytes	Mechanical strain	Panx1/P2X7R-dependent IL-1β priming	Mechanical, not amyloid
Abdullah et al. (2024)	Mouse microglia + astrocytes	P2RX7 loss/inhibition	↓ EV-mediated secretion of pathogenic molecules	—
Diaz-Hernandez et al. (2012)	J20 APP mice, *in vivo*	BBG antagonist	↓ amyloid plaques (GSK3β/secretases)	Pharmacological specificity
Ruan et al. (2020)	P301S tau mice, *in vivo*	GSK1482160 antagonist	↓ EV secretion, improved phenotype	Cell type not dissected
Carvalho et al. (2021)	THY-Tau22 mice, *in vivo*	Genetic P2X7R KO	Improved plasticity/cognition	—
Maschio et al. (2025)	Human AD hippocampus	Quantitative IF	P2X7R enriched in peri-plaque (cored) zone	Correlative
Turcu et al. (2026)	5xFAD mice, *in vivo*	UB-ALT-P2 (brain-penetrant NAM)	↓ cognitive/amyloid/tau/oxidative deficits	Pre-print

### 5.1 eATP amplification as the first inter-glial coupling axis

The first coupling axis links microglia and astrocytes through extracellular ATP itself. Damaged neurons and dystrophic neurites release ATP into the interstitial space (Rodrigues et al., 2015). Microglia detect ATP through multiple purinergic receptors, with high ATP favoring P2X7R activation (Zhang et al., 2020). Activated microglia can then release additional ATP either directly or through mechanisms involving membrane permeabilization and associated channels (Virginio et al., 1999; Chung et al., 2008; Harkat et al., 2017; Karasawa et al., 2017). Astrocytes, in turn, can release ATP via exocytotic routes and through the P2X7R-Panx1 complex (Suadicani et al., 2006; Iglesias et al., 2009). Once this loop is established, glia actively sustain the elevated eATP state, converting a transient neuronal danger signal into a persistent field cue.

This concept is particularly useful in AD, where lesions persist over years rather than hours. A chronic disease requires a chronic signaling loop, and ATP provides a plausible one. In our view, the transition from local neuronal stress to long-lasting glial pathology may depend heavily on whether ATP remains transient or becomes self-sustaining through glial amplification. P2X7R is ideally positioned at both ends of this process. Once established, this sustained eATP environment provides the extracellular trigger for the second axis: the reciprocal relay of inflammatory cytokines and redox signals between microglia and astrocytes.

### 5.2 Cytokine relay: microglial initiation and astrocytic consolidation as a working model

Microglia are the major early producers of IL-1β, IL-18, TNF-α, and ROS after P2X7R activation (Tewari et al., 2024). These mediators are powerful drivers of astrocyte reactivity. Astrocytes exposed to inflammatory cues shift their transcriptional programs, alter transporter expression, change their release profile, and become less effective at supporting synaptic homeostasis (Paumier et al., 2022). Once reactive, astrocytes can themselves produce cytokines and inflammatory mediators, extending and spatially broadening the inflammatory field.

It is important, however, not to imply that astrocytes are obligate downstream responders. Under specific conditions—direct mechanical strain, metabolic or ischemic stress, NAD^+^-dependent ADP-ribosylation that sensitizes P2X7R, or direct exposure to high local concentrations of Aβ or misfolded tau—astrocytic P2X7R and inflammasome-associated pathways can be engaged independently of prior microglial activation, generating IL-1β and reactive astrocyte gene programs cell-autonomously (Hirayama and Koizumi, 2017; Hirayama et al., 2021). We therefore advance the following temporal partitioning as a working hypothesis rather than a settled or unidirectional rule: in the typical AD lesion, microglial P2X7R is likely the earlier responder, converting plaque-associated ATP into cytokine and ROS output, whereas astrocytic P2X7R acts later to consolidate and spatially extend the inflammatory field through gliotransmitter release, reactive gene programs, and further ATP release. In a smaller subset of conditions—where metabolic or mechanical stress reaches astrocytes first, or where astrocytic P2X7R has been sensitized—astrocytes may instead serve as the initiating glial population. This latter possibility is the more speculative, since its supporting evidence derives from ischemic-preconditioning and mechanical-strain paradigms (Albalawi et al., 2017; Hirayama and Koizumi, 2017; Hirayama et al., 2021) rather than amyloid- or tau-bearing tissue; whether it operates in the AD brain is, to our knowledge, untested and constitutes a specific, experimentally addressable prediction of our model—for example, through cell-type-specific or temporally resolved P2X7R manipulation across disease stages.

The receptor thus performs different but mechanistically complementary and bidirectionally coupled jobs in the two cell types: microglia convert ATP into innate immune activation, while astrocytes convert the resulting inflammatory environment into altered network support and extracellular chemistry and—when directly stressed—can themselves drive *de novo* cytokine output. Together, they create the conditions in which neuronal dysfunction becomes progressively harder to reverse. In addition to these soluble mediators, P2X7R-dependent membrane remodeling permits inflammatory and metabolic signals to be packaged into extracellular vesicles, providing the third route of inter-glial propagation.

### 5.3 Extracellular vesicle exchange as a bidirectional glial signaling axis

The role of extracellular vesicles makes the microglia-astrocyte distinction even less separable. Microglial P2X7R activation drives release of IL-1β-containing microvesicles and other extracellular particles (Monif et al., 2016; Weng and Talbot, 2017; van Niel et al., 2018). Astrocytic P2X7R also supports vesicle release under inflammatory conditions (Abdullah et al., 2024). These vesicles may act on neurons, but they may also act on each other's parent glial cell type. A microglial vesicle enriched in inflammatory mediators, lipids, or receptor-associated proteins could alter astrocytic membrane properties or gene expression; an astrocytic vesicle might similarly affect microglial metabolism or receptor responsiveness.

This inter-glial vesicle traffic remains underexplored in AD, yet conceptually it is crucial. It provides a means by which P2X7R signaling can spread through tissue without requiring uniform receptor activation in every cell at the same moment. A lesion core could therefore influence a wider glial territory through packaged messengers, extending the functional radius of ATP-rich pathology. Because these vesicles can also alter neuronal transmitter release and excitability, extracellular vesicle exchange provides a direct mechanistic bridge from inter-glial signaling to the fourth axis, convergent synaptic modulation and circuit destabilization.

### 5.4 Convergent synaptic modulation and circuit destabilization

The fourth coupling axis emerges where microglial and astrocytic P2X7R outputs converge on neuronal function. Microglia-derived extracellular vesicles can enhance excitatory synaptic transmission and alter pre-synaptic release (Gabrielli et al., 2022), whereas astrocytic P2X7R activation can promote glutamate release, influence GABAergic tone, and modify the extracellular ionic and metabolic conditions under which neurons fire (Ficker et al., 2014; Illes et al., 2017). These mechanisms act through different cellular routes but converge on the same functional outcome: loss of stable excitation–inhibition balance and declining synaptic precision.

Neuronal dysfunction can subsequently feed back onto the glial system (Cserép et al., 2020). Hyperactive or metabolically stressed neurons may release additional ATP, increasing extracellular purinergic signaling and further promoting glial P2X7R activation. Neuronal hyperexcitability, ATP release, and glial activation may therefore reinforce one another, allowing circuit dysfunction to persist even without a proportional increase in protein-deposit burden. This neuronal feedback closes the electrophysiological arm of the loop but operates in parallel with a fifth process: the coordinated loss of glial clearance and degradative competence.

### 5.5 Clearance-to-retention: the synchronized housekeeping switch

The fifth coupling axis concerns a coordinated shift in microglial and astrocytic function from uptake, degradation, and tissue containment toward inflammatory secretion and retention or redistribution of pathological material. Both microglia and astrocytes contribute to the removal of extracellular debris and pathogenic proteins in AD (Deng et al., 2024). Microglia phagocytose synaptic elements and plaque-associated material; astrocytes internalize Aβ and certain tau species and process them through lysosomal pathways. P2X7R intersects with these functions in a bidirectional manner. In microglia, the unstimulated receptor can support scavenger activity, whereas activated P2X7R suppresses phagocytosis and favors inflammatory signaling (Gu and Wiley, 2018). In astrocytes, chronic P2X7R activity appears capable of shifting the balance away from efficient autophagic/lysosomal processing toward reactive secretion and stress responses (Kim et al., 2018; Lee and Kim, 2020).

The important consequence is that sustained P2X7R activation may help synchronize a pathogenic switch across both glial populations. Microglia may become less focused on debris uptake and effective degradation and more focused on inflammatory output, whereas astrocytes may become less effective in buffering and lysosomal processing while increasing transmitter release, cytokine production, and extracellular vesicle shedding. This coordinated decline in glial housekeeping defines the clearance-to-retention switch: pathological material is increasingly retained, incompletely degraded, or redistributed rather than effectively contained and removed.

**Figure 4:**
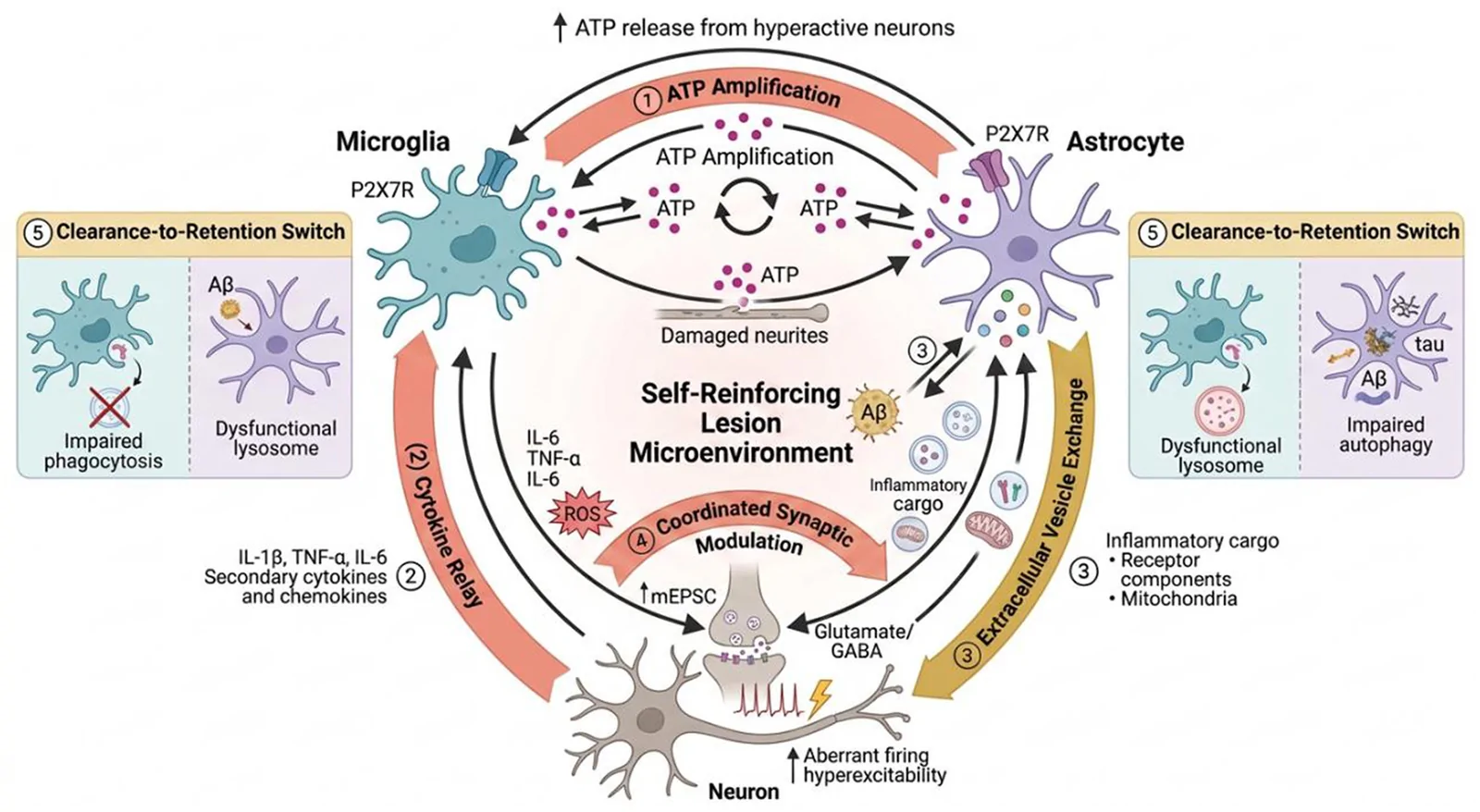
Proposed five-axis P2X7R-mediated reciprocal microglia–astrocyte coupling loop in Alzheimer's disease. Lesion-associated extracellular ATP activates P2X7R in microglia and subsets of astrocytes, initiating a reciprocal signaling loop organized around five interconnected axes. (1) eATP amplification: ATP released from injured neurons and activated glia sustains purinergic signaling and broadens the original danger signal. (2) Cytokine relay: microglial cytokines and reactive oxygen species promote astrocyte reactivity, while reactive astrocytes may further extend the inflammatory extracellular environment. (3) Extracellular vesicle exchange: P2X7R-dependent vesicle release distributes inflammatory, metabolic, and potentially proteopathic cargo among microglia, astrocytes, and neurons. (4) Convergent synaptic modulation: microglial vesicles and astrocytic gliotransmitters alter excitatory and inhibitory neurotransmission. (5) Coordinated clearance-to-retention switch: sustained receptor activation shifts both glial populations away from efficient phagocytic, autophagic, and lysosomal processing and toward inflammatory secretion and retention or redistribution of pathological material. The resulting neuronal hyperexcitability and metabolic stress promote further ATP release, closing the feedback loop. The figure presents an integrative working model: although the individual pathways are supported by experimental evidence, their simultaneous and reciprocal operation as a unified P2X7R-dependent circuit has not yet been directly demonstrated in Alzheimer's disease. eATP, extracellular adenosine triphosphate; ROS, reactive oxygen species; EVs, extracellular vesicles.

Taken together, the five axes describe complementary rather than strictly sequential processes. eATP amplification sustains the extracellular trigger; cytokine relay and extracellular vesicle exchange propagate signals between glial populations; convergent synaptic modulation destabilizes neuronal activity and promotes further ATP release; and the coordinated clearance-to-retention switch weakens the capacity of the tissue to contain pathogenic proteins and cellular debris. Figure 4 integrates these processes into a single reciprocal circuit. The sections that follow locate this circuit within the amyloid plaque niche (Section 6) and examine its two principal downstream consequences: synaptic dysfunction (Section 7) and proteostatic failure (Section 8).

## 6 P2X7R within the amyloid plaque niche: a cross-glial model of local disease amplification

Having assembled the five-axis loop in Section 5, we now ask where in the AD brain it is spatially concentrated. Beyond serving as histopathological landmarks, Aβ plaques act as organizers of a pathological microenvironment. The tissue surrounding plaques constitutes a metabolically and immunologically active niche in which damaged neurites, altered synaptic activity, eATP accumulation, microglial activation, and astrocytic reactivity coexist (Mallach et al., 2024). In this niche, P2X7R is particularly well placed to coordinate local disease amplification.

Microglia are typically the first glial population to accumulate around plaques. They recognize protein aggregates and damaged tissue through innate immune receptors and can transition toward DAM-like states involving altered lipid metabolism, lysosomal activity, and phagocytosis (Cheng and Ho, 2025). This response is not inherently harmful; indeed, plaque-associated microglia may initially protect the surrounding neuropil by physically isolating aggregates and attempting their removal (Zhong et al., 2018; Jain et al., 2023). However, the plaque niche also contains multiple potential sources of extracellular ATP, including stressed neurons, dystrophic neurites, and activated glia (Rodrigues et al., 2015). Under such conditions, microglial P2X7R introduces a second layer of activation. The result is not simply enhanced surveillance, but a shift toward inflammasome activity, ROS generation, vesicle shedding, and reduced scavenger efficiency (Tewari et al., 2024).

Astrocytes are recruited into the same niche, where they surround plaques, alter their metabolic state, and modify transporter and gliotransmitter systems (Andersen et al., 2022). In APP/PS1 mice, plaque-associated astrocytes show reduced excitatory amino acid transporter 2 (EAAT2) in some studies, favoring extrasynaptic glutamate accumulation and neuronal hyperactivity (Li et al., 2011; Hefendehl et al., 2016; Smit et al., 2021), though human tissue data suggest that astrocytes may also mount compensatory increases in EAAT2 under certain pathological conditions (Kobayashi et al., 2018). This discrepancy likely reflects a disease stage issue: astrocytes may initially adapt, then later fail. P2X7R offers a plausible mechanism for this transition. As ATP rises and inflammatory signals from microglia accumulate, astrocytic P2X7R can enhance glutamate release, promote reactive gene programs, and potentially weaken degradative pathways (Beltran-Lobo et al., 2023).

**Figure 5:**
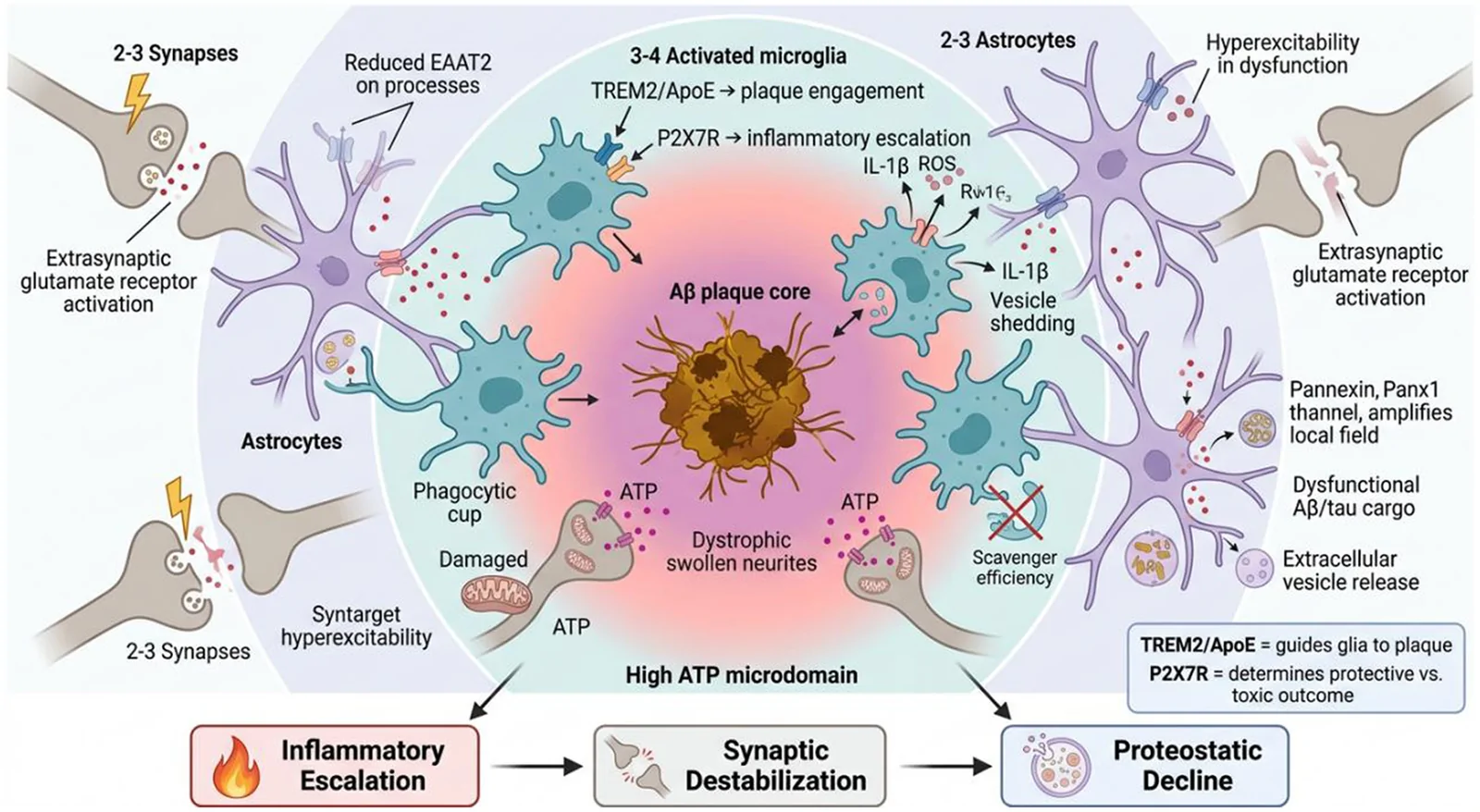
Proposed amyloid plaque niche as a spatially restricted P2X7R-sensitive ATP microdomain. Compact or cored amyloid-β (Aβ) plaques are surrounded by dystrophic neurites, metabolically stressed synapses, activated microglia, and reactive astrocytes, all of which may serve as local sources of extracellular ATP (eATP). Human Alzheimer's disease tissue analyses have identified increased P2X7R immunoreactivity within the peri-plaque region surrounding compact plaques, whereas comparable enrichment was not observed around diffuse plaques. In the proposed model, TREM2- and ApoE-associated signaling initially supports microglial recruitment, plaque engagement, and containment. As lesion-associated stress and eATP increase, P2X7R signaling may superimpose a high-ATP stress program that promotes cytokine production, oxidative stress, extracellular vesicle release, and reduced clearance competence. Astrocytes recruited into the same niche may undergo P2X7R-associated changes in ATP release, gliotransmission, inflammatory signaling, transporter function, and lysosomal processing. Reciprocal signaling between the two glial populations is proposed to produce three convergent outcomes: inflammatory escalation, synaptic destabilization, and declining proteostatic capacity. Importantly, eATP concentrations have not yet been directly measured within the peri-plaque region in Alzheimer's disease tissue. The designation of this region as a P2X7R-sensitive ATP microdomain therefore represents a spatially defined and testable model rather than a directly demonstrated biochemical microenvironment. Aβ, amyloid-β; eATP, extracellular adenosine triphosphate; TREM2, triggering receptor expressed on myeloid cells 2; ApoE, apolipoprotein E; EVs, extracellular vesicles.

The role of ApoE and TREM2 in the plaque niche also intersects conceptually with P2X7R. ApoE is both a major AD risk factor and a regulator of glial handling of Aβ (Liu et al., 2025). TREM2 drives aspects of microglial plaque engagement and DAM conversion (Poliani et al., 2015; Wang et al., 2022). Yet neither pathway by itself explains why plaque-associated glia become progressively inflammatory or why neighboring synapses become dysfunctional even when plaques are still being partially contained. P2X7R may fill this explanatory gap by linking lesion-associated ATP accumulation to a more destructive glial phenotype. In other words, and consistent with the distinction drawn in Section 3, TREM2 and ApoE may direct glial recruitment and plaque engagement, whereas P2X7R may determine whether peri-plaque glia retain containment-associated functions or shift toward injury-promoting inflammatory and secretory activity (Figure 5).

A similar logic applies to tau pathology. Astrocytes can internalize extracellular tau, including fibrillar species, and direct them toward lysosomal degradation, while HSPGs and LRP1 contribute to tau uptake (Holmes et al., 2013; Merezhko et al., 2018). Microglia also participate in tau-related inflammatory remodeling. If P2X7R activation simultaneously impairs degradative efficiency and enhances secretory/inflammatory behavior, then the receptor may contribute to the spread of tau pathology indirectly by making glial cells less capable of processing what they take up. Direct *in vivo* evidence supports this: in P301S tau-transgenic mice, the P2X7R antagonist GSK1482160 suppressed exosome/EV secretion from glia and improved pathology and behavior (Ruan et al., 2020), and genetic P2X7R deletion in the THY-Tau22 tauopathy model improved hippocampal plasticity and cognition (Carvalho et al., 2021). On the amyloid side, *in vivo* pharmacological P2X7R inhibition (Brilliant Blue G) in J20 APP mice reduced plaque load via GSK3β/secretase-linked mechanisms. These convergent genetic and pharmacological rodent studies strengthen causal interpretation, though cell-type-specific contributions were not fully dissected. Recent quantitative analyses of human AD hippocampal tissue provide spatial support for this model while also emphasizing substantial cell-type heterogeneity (Maschio et al., 2025). P2X7R immunoreactivity was significantly enriched within the peri-plaque region, operationally defined as the area extending approximately 50 μm from the core of compact or cored Aβ plaques, whereas comparable enrichment was not observed around diffuse plaques. In the hippocampal regions examined, P2X7R showed substantially greater colocalization with GFAP-positive astrocytes than with the microglial markers Iba1, TMEM119, P2Y12R, or CD68, indicating a pre-dominantly astrocytic localization in this tissue context. P2X7R was nevertheless detectable in subsets of microglia and was positively associated with Aβ and phosphorylated tau pathology. These findings support a glial and plaque-associated distribution of P2X7R but do not establish the relative functional contributions of astrocytic and microglial receptors or demonstrate reciprocal signaling between the two cell populations. Moreover, because local eATP concentrations were not measured, the observed peri-plaque enrichment of P2X7R should be interpreted as spatial support for, rather than direct validation of, the proposed P2X7R-sensitive ATP microdomain.

For these reasons, we propose—as a spatially defined but as-yet-unproven model—that the plaque niche may function as a P2X7R-sensitive ATP microdomain. By “microdomain” we mean specifically the peri-plaque zone, operationally the region within roughly 50 μm of a compact, cored Aβ deposit in which P2X7R immunoreactivity is enriched in human AD tissue (Maschio et al., 2025), and within which dystrophic neurites, metabolically stressed synapses, activated microglia, and reactive astrocytes constitute local ATP sources (Rodrigues et al., 2015). We note an important gap in this account: to our knowledge, eATP has not been directly measured in this zone in AD tissue, and it therefore remains an inference—not a demonstrated fact—that local ATP reaches the high-micromolar-to-millimolar concentrations (i.e., approaching the several-hundred-μm-to-~1 mm EC50 defined in Section 2) required to activate P2X7R's low-affinity binding site. Establishing this is a pre-requisite for the model and a priority for future work. Within this proposed microdomain, we hypothesize that microglia and astrocytes may participate in a locally self-reinforcing program involving inflammatory escalation, synaptic destabilization, and declining proteostatic competence. These three outcomes are often studied separately; our model predicts that in AD they are causally coupled, but we stress that this coupling is inferred from convergent indirect evidence rather than directly demonstrated, and that the alternative—that the three arise in parallel from shared upstream pathology without reciprocal reinforcement—is not excluded by current data.

## 7 Synaptic dysfunction as the shared downstream consequence of glial P2X7R activation

The loop assembled above matters clinically only if it reaches the synapse. This section focuses primarily on the fourth axis—convergent synaptic modulation and circuit destabilization—while also considering the indirect contribution of the cytokine-relay axis. Because memory failure in AD tracks closely with progressive loss of synaptic plasticity, a receptor confined to glial inflammation would relate to cognition only indirectly. P2X7R, however, converges on synaptic physiology through several glial routes, which is why the coupling model—not isolated receptor biology—best explains its cognitive relevance.

Microglia contribute to synaptic remodeling during development and adulthood, and in disease they can aberrantly eliminate or destabilize synapses (Stowell et al., 2019; Wang et al., 2020). Extracellular vesicles released from P2X7R-activated microglia increase excitatory post-synaptic event frequency (Kanno et al., 2010; Norman et al., 2010), suggesting that inflammatory microglia can directly bias pre-synaptic behavior. In AD, such effects could reinforce neuronal hyperactivity already associated with early amyloid pathology. Hyperactive neurons release more ATP and impose greater metabolic demand on astrocytes, further tightening the cross-glial loop.

Astrocytes regulate the extracellular concentration of glutamate, GABA, and other signaling molecules that determine whether synaptic activity remains spatially and temporally precise (Kruyer et al., 2023; Purushotham and Buskila, 2023). P2X7R-dependent glutamate release by astrocytes has been demonstrated in culture and slices (Zhao et al., 2021), and P2X7R-related astrocytic GABA signaling has also been described (Khan et al., 2019). In AD, these mechanisms could have two distinct but related consequences. First, they may promote aberrant neuronal firing and extrasynaptic receptor activation in plaque-rich territories. Second, they may mask the primary lesion by introducing secondary circuit noise that exceeds what would be expected from protein deposition alone.

Metabolic support to synapses is also relevant. Astrocytes are essential providers of metabolic intermediates and contribute to L-serine availability, which in turn influences NMDA receptor-dependent plasticity through neuronal D-serine production (Mosconi, 2005). Because AD disrupts astrocytic metabolism early (An et al., 2018; Bonvento et al., 2022), receptor systems that further shift astrocytes from homeostatic support to reactive secretion may have profound indirect synaptic effects. P2X7R is a likely participant in this shift, particularly under ATP-rich conditions where the receptor drives gliotransmitter release and inflammatory programs.

Glial P2X7R therefore contributes to the fourth coupling axis through several distinct routes rather than through inflammation alone. Microglial P2X7R modifies the cytokine and extracellular-vesicle content of the interstitial space, whereas astrocytic P2X7R alters extracellular glutamate and GABA concentrations, ion buffering, and metabolic support. Together these actions modify the extracellular conditions that govern synaptic transmission and plasticity. This may explain why genetic deletion or pharmacological inhibition of P2X7R improves long-term synaptic plasticity, spatial learning, and memory in AD-related models (Diaz-Hernandez et al., 2012). The benefit is unlikely to derive from one single downstream pathway. It may instead reflect interruption of the broader five-axis feedback system in which inflammatory signaling, extracellular vesicle release, gliotransmission, neuronal hyperexcitability, and further ATP release progressively erode synaptic resilience.

## 8 Protein clearance and proteostasis: the overlooked cross-glial dimension of P2X7R in AD

We turn finally to the fifth coupling axis, the coordinated clearance-to-retention switch, which is among the least studied but potentially most consequential components of the proposed loop for long-term disease progression. Inflammation and synaptic dysfunction are obvious features of AD, but the disease also reflects failure of tissue-level proteostasis. Protein aggregates persist not only because they are generated, but because the cells tasked with containing, internalizing, processing, and clearing them become progressively less effective. Microglia and astrocytes share this burden in different ways, and P2X7R may influence both.

Microglial phagocytosis is often described as beneficial in AD, but this is only partially true. It is beneficial when it remains coupled to effective degradation and controlled inflammatory output. Once uptake becomes inefficient, lysosomal stress rises, or phagocytosis is suppressed while cytokine secretion remains high, microglia may worsen the local lesion (Fabbrizio et al., 2017; Sekar et al., 2018). Because ATP-activated P2X7R can suppress scavenger-type phagocytosis and impair degradative competence through lysosomal alkalinization, the receptor offers a mechanistic route by which microglia lose protective efficiency within ATP-rich plaque territories.

Astrocytes play a parallel but underappreciated role. Mature astrocytes internalize Aβ *in vitro* and *ex vivo* (Lasagna-Reeves and Kayed, 2011), and astrocytic uptake of amyloid depends on ApoE, LRP1, and TFEB-linked lysosomal function (Xiao et al., 2014). Astrocytes also express extracellular proteases capable of degrading Aβ (Leal et al., 2006). Tau handling follows a similarly complex logic: astrocytes can internalize extracellular tau and direct it to lysosomes, yet under some circumstances they may also contribute to the spread of tau species (Martini-Stoica et al., 2018; Puangmalai et al., 2020; Rauch et al., 2020). These data establish one point unequivocally: astrocytes are active participants in proteostasis.

The contribution of P2X7R to astrocytic proteostasis is still insufficiently mapped, but several clues are significant. P2X7R influences autophagy in astrocytes (Kim et al., 2018; Lee and Kim, 2020), and signaling through focal adhesion kinase and small heat-shock proteins has been tied to receptor-dependent control of autophagic pathways (Kim et al., 2018; Lee and Kim, 2020). In other tissues, P2X7R can regulate HSPG expression and localization (Mankus et al., 2012), a notable finding given the importance of HSPGs in tau uptake and release (Holmes et al., 2013; Merezhko et al., 2018). These observations suggest that receptor activation may influence not only whether astrocytes become inflammatory, but whether they remain competent to process pathogenic proteins rather than redistribute them.

This may be one of the most important reasons why P2X7R inhibition appears beneficial in amyloidosis and tauopathy models (Carvalho et al., 2021). The usual interpretation is that reduced cytokine release lessens damage. That is likely correct, but incomplete. A second possibility is that suppressing P2X7R helps preserve the degradative and buffering roles of glia, thereby improving the overall capacity of the tissue to handle pathological proteins. In our view, this proteostasis-centered interpretation deserves more attention. AD is a disease in which inflammation and clearance failure are closely associated. We therefore propose—while emphasizing that this remains to be demonstrated directly—that P2X7R may help connect inflammatory activation to the fifth coupling axis, in which microglial and astrocytic housekeeping progressively shifts from effective clearance toward retention, incomplete degradation, or redistribution of pathological material.

## 9 Therapeutic implications: why a cross-glial view of P2X7R matters

The therapeutic appeal of P2X7R is obvious: it is activated preferentially in pathological ATP environments, is strongly linked to inflammatory signaling, and has shown beneficial responses to antagonism or genetic deletion in AD-related models (Donnelly-Roberts and Jarvis, 2007). Yet the receptor has also proven difficult to translate straightforwardly into clinical success. One reason may be that it has too often been targeted as if it were a generic inflammation switch. A cross-glial view suggests a more nuanced strategy organized around six considerations: pharmacological selectivity, central nervous system exposure, cell-type and disease-stage specificity, species-aware translational design, function-oriented biomarkers, and rational combination therapy.

### 9.1 Pharmacological selectivity: state-dependent and allosteric inhibition

Not all P2X7R functions are equally undesirable. The unstimulated or closed conformation may support scavenger-like phagocytic behavior important for debris clearance (Gu and Wiley, 2018). Indiscriminate blockade across receptor states could therefore suppress a protective aspect of glial housekeeping. The therapeutic ideal is to inhibit the sustained, high-ATP, inflammasome-associated and membrane-remodeling functions of P2X7R while sparing receptor configurations that support scavenger activity or controlled surveillance. The existence of non-orthosteric inhibitory sites raises the possibility that such selectivity is pharmacologically feasible (Karasawa and Kawate, 2016; Allsopp et al., 2017).

Recent structural work has shown that this kind of selectivity is now within reach. High-resolution cryo-EM analysis of the rat P2X7R bound to six chemically diverse allosteric antagonists revealed at least three distinct modes of allosteric inhibition: shallow binders (e.g., A438079, AZD9056, GSK1482160), deep binders (e.g., JNJ47965567, which extends into the classical allosteric pocket and forms inter-ligand contacts), and a newly described “starfish” mode in which the previously unidentified antagonist methyl blue simultaneously engages an extended allosteric site composed of three sub-pockets and forms cooperative inter-ligand interactions across the threefold symmetry axis (Oken et al., 2024a). This structural taxonomy provides a molecular blueprint for tuning subtype selectivity, residence time, and even multivalent ligand design. Building directly on this framework, Oken et al. (2025) demonstrated that such selectivity is empirically achievable in the human receptor: leveraging cryo-EM structures of the human, mouse, and rat orthologs, they identified a polycyclic scaffold compound, UB-MBX-46, that engages the classical allosteric pocket of the human P2X7R with sub-nanomolar potency, high subtype selectivity over other P2XRs, and a near-irreversible binding profile. This kinetic feature is particularly suited to the chronically ATP-rich microenvironment of the AD brain, where short-acting inhibition would be quickly overwhelmed by sustained ligand exposure.

### 9.2 From structural insight to brain-penetrant therapeutics

Translating these structural insights into a clinically relevant compound requires not only target selectivity but also adequate central nervous system exposure—a hurdle that has limited several earlier P2X7R antagonists. Turcu et al. (2026) recently addressed this challenge with UB-ALT-P2, a brain-penetrant negative allosteric modulator that engages P2X7R with sub-nanomolar potency and prolonged target residence time. In the 5xFAD mouse model of AD, UB-ALT-P2 simultaneously attenuated cognitive deficits, amyloid burden, tau pathology, oxidative stress, and neuroinflammatory readouts at well-tolerated doses with confirmed blood-brain barrier penetration and central target engagement.

This pleiotropic efficacy profile is consistent with, but does not on its own establish, the framework proposed in this review. We caution against over-reading it. The fact that a single high-threshold antagonist can relieve cognitive, proteinopathic, oxidative, and inflammatory phenotypes simultaneously is compatible with interruption of a shared upstream coupling node, but it does not distinguish that interpretation from at least two simpler alternatives: (i) a linear cascade in which P2X7R sits upstream of sequentially dependent events, and (ii) the possibility that the several readouts reflect correlated disease severity that improves together whenever any dominant driver is attenuated. Broad efficacy of one upstream antagonist cannot, by itself, adjudicate between a coupling hub and a linear pathway; distinguishing them would require, for example, cell-type-specific P2X7R deletion or interventions that dissociate the individual axes. In other words, UB-ALT-P2 is consistent with—rather than confirmatory of—the interpretation of P2X7R as a cross-glial coupling node: when microglia–astrocyte amplification, synaptic destabilization, and proteostatic failure are mechanistically entangled through ATP-driven feedback, focused inhibition of their common transducer can produce broader benefits than would be expected from targeting any single downstream pathway. From a translational standpoint, the emergence of compounds combining sub-nanomolar potency, allosteric selectivity, near-irreversible binding kinetics, and brain penetrance strengthens the case for P2X7R as a tractable candidate target in AD and supports further evaluation of its therapeutic potential.

### 9.3 Cell-type and disease-stage specificity

In early AD, when the dominant signal is plaque-associated ATP elevation and microglial inflammasome priming, preventing P2X7R-driven inflammatory escalation may be the immediate priority. At later stages, when astrocytic homeostatic capacity has already begun to fail, preserving astrocytic glutamate buffering, protein degradation, and metabolic support may be equally important. If both compartments are targeted identically across disease stages, opportunities for stage-specific intervention may be missed. The cross-glial framework predicts that high-threshold antagonists with sustained brain exposure—such as UB-ALT-P2—should be most effective in disease windows where ATP-driven amplification is actively converting transient injury into self-sustaining lesions, namely the prodromal and early symptomatic phases when synaptic dysfunction is established but neurodegeneration remains partially reversible.

### 9.4 Species-aware translational design

Translational interpretation of P2X7R pharmacology requires careful attention to species differences. Some P2X7R-dependent phenomena observed in rodent microglia are attenuated or absent in cultured human microglia (Janks et al., 2018, 2019), and human splice variants differ from rodent forms (Cheewatrakoolpong et al., 2005; Liang et al., 2015; Sluyter, 2017). Even single-residue divergences between human and rodent receptors can substantially alter antagonist binding kinetics (Turcu et al., 2026), helping to explain why several rodent-validated antagonists have shown attenuated effects in human-cell systems and earlier clinical trials. Astrocyte heterogeneity adds another layer of complexity, since regional and disease-stage differences in astrocytic P2X7R expression may shape both efficacy and tolerability. For these reasons, translational pipelines should rely less on simplified monoculture assumptions and more on human-centered co-culture systems, induced pluripotent stem cell-derived microglia and astrocyte models, organoid platforms, and spatial transcriptomic analyses of diseased tissue. The cross-species structural validation across human, mouse, and rat orthologs adopted by Oken et al. (2025) provides a useful template for de-risking species translation early in the discovery pipeline.

### 9.5 Function-oriented biomarkers: extracellular vesicles and cytokines as readouts of P2X7R-driven pathology

Biomarkers should reflect receptor function rather than expression alone. Measuring P2X7R abundance may identify reactive tissue but does not distinguish between receptor states, the local ATP microenvironment, or whether the dominant downstream effect is cytokine release, vesicle shedding, or proteostatic failure. Two functional readouts are particularly informative for the cross-glial framework proposed in this review.

First, P2X7R is a master regulator of glial extracellular vesicle (EV) biogenesis. As discussed in Sections 3 and 4, sustained receptor activation drives microvesicle shedding and mitochondria-containing EV release from microglia and astrocytes, carrying inflammatory cargo such as IL-1β, NLRP3 components, and oxidized mtDNA into the interstitial space and, ultimately, into accessible biofluids (Abdullah et al., 2024; Falzoni et al., 2024). Because EV release scales with receptor engagement, longitudinal profiling of microglia- and astrocyte-derived EVs in plasma or CSF could provide a dynamic pharmacodynamic readout of P2X7R activity *in vivo*—a capability that static expression-based imaging alone cannot offer.

Second, downstream cytokines—particularly IL-1β and IL-18, together with their balance against anti-inflammatory mediators—constitute a quantitative pharmacodynamic signature of the P2X7R–NLRP3 axis (Ferrari et al., 2006; Savio et al., 2018). Combining EV profiling, cytokine panels, and complementary modalities such as P2X7R PET imaging or transcript quantification may offer the most reliable strategy to (i) identify the disease stage at which P2X7R-targeted therapy is most likely to be efficacious, (ii) confirm central target engagement in early-phase trials of brain-penetrant antagonists such as UB-ALT-P2 (Turcu et al., 2026), and (iii) stratify patients by inflammatory phenotype rather than by clinical or amyloid status alone. This biomarker-driven logic mirrors the role that amyloid PET and plasma p-tau217 now play in stratifying patients for anti-Aβ immunotherapy trials (Cummings et al., 2025), supporting the feasibility of analogous fluid-based stratification for inflammation-targeted interventions.

### 9.6 Combination therapy: P2X7R blockade as a complement to anti-Aβ and anti-tau disease-modifying therapies

The recent approval of anti-Aβ monoclonal antibodies—lecanemab (van Dyck et al., 2023) and donanemab (Sims et al., 2023)—marks a milestone in AD therapeutics, yet their clinical benefit remains modest and is partially offset by amyloid-related imaging abnormalities (ARIA), which involve neurovascular inflammation and perivascular microglial activation during antibody-mediated plaque clearance. Anti-tau immunotherapies and active vaccines currently in clinical evaluation similarly target proteinopathy but do not directly resolve the chronic neuroinflammatory milieu that sustains synaptic loss and tau propagation. Notably, the 2025 AD drug development pipeline contains 138 agents across 182 active trials, of which roughly 17% target neuroinflammation/immune processes, and combination regimens are now an explicit feature of the pipeline (Cummings et al., 2025). This therapeutic landscape provides a concrete clinical context within which the cross-glial role of P2X7R can be operationalized.

In this context, P2X7R antagonism offers a mechanistically complementary strategy rather than a competing one. Because P2X7R sits upstream of NLRP3 inflammasome priming, IL-1β/IL-18 maturation, microvesicle shedding, and microglial pyroptotic-like signaling (Savio et al., 2018), its blockade is predicted to (i) attenuate ARIA-associated neurovascular inflammation triggered by antibody-mediated amyloid mobilization, (ii) enhance microglial phagocytic competence toward antibody-opsonized Aβ plaques by preventing the ATP-driven scavenger-to-inflammatory functional switch (Gu and Wiley, 2018; Campagno et al., 2021), and (iii) limit tau propagation via inflammasome-dependent EV release from glia (Ruan et al., 2020). Pre-clinical evidence supports this rationale: in amyloidosis and tauopathy models, P2X7R antagonism or genetic deletion reduces plaque burden, tau hyperphosphorylation, microglial inflammasome activity, and behavioral deficits (Ruan et al., 2020; Carvalho et al., 2021; Turcu et al., 2026).

Conceptually, this combinatorial logic aligns with the cross-glial framework proposed here. Anti-Aβ and anti-tau DMTs target the substrate of pathology (the misfolded protein), whereas we argue that P2X7R blockade targets the transducer through which proteinopathy may be converted into chronic, self-reinforcing glial dysfunction. The two strategies therefore act on non-redundant nodes of AD pathogenesis. We propose that future clinical trials evaluate rationally designed combination regimens pairing next-generation CNS-penetrant P2X7R antagonists (Turcu et al., 2026) with approved or investigational anti-Aβ/anti-tau DMTs (Sims et al., 2023; van Dyck et al., 2023), prospectively incorporating EV- and cytokine-based readouts (Section 9.5) as secondary endpoints. Such a precision-intervention framework would allow enrichment of trials for patients with a high “P2X7R-active” inflammatory phenotype, longitudinal monitoring of pharmacodynamic response, and rational dose titration in combination regimens—repositioning P2X7R from a purely pharmacological target into a biomarker-defined therapeutic axis at the intersection of proteinopathy and neuroinflammation.

## 10 Conclusion

The role of P2X7R in AD is often reduced to a simple formula in which eATP activates microglia, triggers inflammasome assembly, and promotes neuroinflammation. Although this sequence is valid, it captures only a fraction of the receptor's significance. In the AD brain, P2X7R is better understood as a mechanism of cross-glial state conversion. It translates lesion-associated ATP accumulation into coordinated changes in microglia and astrocytes, and these changes converge on the three processes that most strongly determine disease progression: inflammatory amplification, synaptic destabilization, and defective protein clearance. Within this framework, the proposed coupling process is organized in the order summarized in Figure 4, lesion-associated eATP is sustained through the eATP-amplification axis; inflammatory signals propagate through cytokine relay; packaged mediators extend the response through extracellular vesicle exchange; convergent microglial and astrocytic effects destabilize synaptic and circuit function; and declining phagocytic, autophagic, and lysosomal competence produces a coordinated clearance-to-retention switch.

Microglial and astrocytic P2X7R may make complementary contributions across the five coupling axes. Both glial populations can contribute to maintenance of an ATP-rich extracellular environment; cytokine and ROS production supports inflammatory relay; microvesicle and extracellular-vesicle release extends signaling beyond the initially activated cell; microglial vesicles and astrocytic gliotransmitters converge on synaptic and circuit function; and declining phagocytic, autophagic, and lysosomal competence promotes a coordinated shift from clearance toward retention. Although microglia may act as the earlier responder in many plaque-associated contexts, the sequence is unlikely to be universal, and directly stressed or sensitized astrocytes may also initiate components of the loop. These two receptor programs are not parallel. They are reciprocal. Microglial P2X7R provides the initial inflammatory output, while astrocytic P2X7R sustains the altered state through metabolic, synaptic, and extracellular changes, together maintaining a self-reinforcing lesion microenvironment.

This interpretation also helps explain why P2X7R antagonism improves synaptic plasticity, memory, and pathological burden in AD models (Ruan et al., 2020). The receptor is not valuable as a target simply because it reduces IL-1β. It is valuable because it sits at the point where ATP-rich pathology becomes a multicellular feedback loop. Breaking that loop may preserve both neuronal function and glial competence.

In our view, future work should move beyond asking whether P2X7R is expressed in one glial population or another and instead define how receptor activity is partitioned across microglia-astrocyte signaling axes in different stages of AD. That is the question most likely to yield mechanistic clarity and therapeutic leverage. If AD is indeed a disease of failed multicellular coordination around pathological protein stress, then P2X7R is not a peripheral player. It is one of the receptors through which that failure is organized.
